# Quantitative Muscle MRI Fat Fraction as a Biomarker of Disease Severity in Mitochondrial Myopathies

**DOI:** 10.1002/jcsm.70380

**Published:** 2026-09-15

**Authors:** Ana Bermejo‐Moriñigo, Paloma Martín‐Jiménez, Violeta González‐Méndez, Laura Bermejo‐Guerrero, Luz Edith Ochoa, Cristina Martín‐Arriscado, Andrea Alcalá‐Galiano, Cristina Casado‐Pérez, María Navarro‐Riquelme, Rocío Garrido‐Moraga, Adrián González Quintana, Alberto Blázquez, Cristina Domínguez‐González

**Affiliations:** ^1^ Radiology Department Hospital Universitario 12 de Octubre Madrid Spain; ^2^ Neuromuscular Diseases Unit, Neurology Department Hospital Universitario 12 de Octubre Madrid Spain; ^3^ Medical‐Surgical Sciences Research, Faculty of Medicine Complutense University of Madrid Madrid Spain; ^4^ Mitochondrial and Neuromuscular Research Group ‘12 de Octubre’ Instituto de Investigación Hospital Universitario 12 de Octubre (imas12) Madrid Spain; ^5^ Unidad de Investigación y Soporte Científico Instituto de Investigación Hospital Universitario 12 de Octubre (imas12) Madrid Spain; ^6^ Biomedical Network Research Centre on Rare Diseases (CIBERER), Instituto de Salud Carlos III Madrid Spain

**Keywords:** biomarkers, mitochondrial myopathies, MRI‐derived fat fraction, quantitative muscle MRI

## Abstract

**Background:**

Quantitative muscle MRI is increasingly used to assess structural muscle damage in inherited myopathies, but its application in primary mitochondrial myopathies (PMM) has not been systematically evaluated in large cohorts. Because PMM are clinically and genetically heterogeneous, objective imaging biomarkers are needed to quantify skeletal‐muscle involvement and define meaningful subgroups. We assessed whether MRI‐derived proton density fat fraction (PDFF) captures genotype‐ and phenotype‐specific patterns of fatty replacement and reflects disease severity using motor function outcomes and circulating biomarkers.

**Methods:**

We performed a cross‐sectional analysis of 49 adults with genetically confirmed PMM enrolled in a prospective natural history study. Participants underwent standardised lower‐limb MRI, including T1‐weighted sequences and mDIXON‐QUANT fat fraction imaging. Muscle involvement was assessed semi‐quantitatively with the modified Mercuri scale and quantitatively by PDFF measurement in selected pelvic girdle and thigh muscles. A total fat fraction score (FF sum) was calculated. PDFF was compared across genotypes and phenotypes and against MRC score, NSAA, 6‐min walk test, 100‐m run test, CK, serum creatinine, GDF15 and disease duration.

**Results:**

Mean age at onset was 26.3 ± 15.1 years, mean age at MRI was 50.0 ± 12.3 years, and median disease duration was 23 years (IQR 15–31). Genetic diagnoses included mtDNA variants in 29 patients (59.1%; single large‐scale deletions, *n* = 19; point mutations, *n* = 10), nuclear variants in *POLG* (*n* = 8), *TK2* (*n* = 8) and *TWNK* (*n* = 4). Phenotypes were exercise intolerance without overt weakness (*n* = 7), isolated progressive external ophthalmoplegia (PEO; *n* = 16), PEO‐plus (*n* = 16) and progressive myopathy (*n* = 8). Highest median PDFF values were observed in *tensor fasciae latae* (31%), *gluteus maximus* (30%), *sartorius* (25%) and *gracilis* (20%). FF sum correlated with MRC score (*r* = −0.567, *p* < 0.0001), NSAA (*r* = −0.731, *p* < 0.0001), 100‐m run test time (*r* = 0.629, *p* < 0.0001), 6‐min walk distance (*r* = −0.311, *p* = 0.0476) and serum creatinine (*r* = −0.715, *p* < 0.0001), but not with CK or GDF15. TK2 deficiency showed the highest fat replacement, particularly in *gluteus maximus* and *gracilis* (*p* < 0.001). Progressive myopathy showed greater fatty replacement than other phenotypes (*p* < 0.001), while exercise intolerance showed higher fat fraction than isolated PEO (*p* = 0.033).

**Conclusions:**

Quantitative muscle MRI provides objective and clinically meaningful measures of muscle involvement in PMM. MRI‐derived fat fraction reflects disease severity, discriminates between genotypes and phenotypes, and detects subclinical muscle damage. These cross‐sectional findings support PDFF as a candidate imaging biomarker for patient stratification and disease characterisation; prospective longitudinal studies will be required to establish its sensitivity to change and its validity as an outcome measure for therapeutic trials.

## 1 Introduction

Mitochondrial myopathies are a clinically and genetically heterogeneous group of disorders caused by pathogenic variants in mitochondrial DNA (mtDNA) or in nuclear genes (nDNA) involved in mitochondrial DNA replication and maintenance [1]. This heterogeneity results in wide variability in clinical presentation, disease severity and progression, complicating patient stratification and the selection of appropriate outcome measures [2]. With the development of disease‐modifying therapies, there is a growing need for reliable biomarkers that enable accurate assessment of disease burden, identification of homogeneous patient subgroups and optimisation of patient selection for clinical trials [3, 4]. Muscle magnetic resonance imaging (MRI) is an established tool in the evaluation of many inherited muscle diseases [5, 6], providing diagnostic pattern recognition and objective measures of disease severity through the assessment of muscle fatty replacement [7, 8, 9]. However, in mitochondrial myopathies, the clinical utility of muscle MRI remains less well defined and quantitative imaging data are limited [10]. Advances in quantitative MRI techniques, including fat fraction analysis, offer the potential to provide reproducible, clinically meaningful biomarkers of muscle involvement. In particular, proton density fat fraction (PDFF) mapping, derived from chemical shift encoding‐based water‐fat MRI, has emerged as the most reliable technique for quantifying fat infiltration in muscle tissue, providing high accuracy, reproducibility and robustness against acquisition parameter variations [11, 12]. The aim of this study was to assess the diagnostic value of PDFF in correlation with the well‐established semi‐quantitative Mercuri grading [13] in a well‐characterised cohort of adults with primary mitochondrial myopathies and to examine its association with clinical phenotypes, genotypes, functional measures and circulating biomarkers.

## 2 Methods

### 2.1 Patients and Study Setup

We prospectively collected clinical data, functional motor outcome measures and biomarkers in adult patients diagnosed with PMM due to pathogenic mtDNA or nDNA variants, followed at a single Spanish Reference Center for Neuromuscular and Mitochondrial Myopathies, within a Natural History Study. The study was registered on ClinicalTrials.gov with ID NCT05653544 in December 2022 and the first patient enrolled signed informed consent in February 2023. Disease duration was defined as the interval between symptom onset and MRI evaluation.

### 2.2 Clinical Examination‐Motor Function Assessment

The motor function was assessed by the Motor Research Council scale (MRC). MRC grades muscle strength on a scale from 0 to 5, with each grade representing a specific level of muscle contraction and functional ability, and the average strength measured in 10 muscles has been calculated; the 6‐min walk test (6MWT), the North Star Ambulatory Assessment (NSAA), which evaluates motor goals with a score range of 0 (minimum) to 34 (maximum), and the 100‐m run test (100MRT), that measures the time to cover 100 m at the highest possible speed.

### 2.3 Biomarkers

Serum lactate, creatine kinase (CK) and creatinine levels were measured by standard clinical laboratory protocols and compared with reference ranges at Hospital 12 de Octubre (lactate: 0.5 and 2.2 mmol/L, CK: 34–171 U/L, creatinine: 0.7 and 1.2 mg/dL). Circulating GDF‐15 was assessed in plasma by a quantitative electrochemiluminescence immunoassay (Elecsys GDF‐15; Roche Diagnostics, Basel, Switzerland). Reference values (pg/mL) were provided by the kit's manufacturer, according to age (median, 95th percentile): 30–40 (500, 852), 40–50 (614, 1229), 50–60 (757, 1466), 60–70 (866, 1476), >70 (1060, 2199). The performance and validation of that GDF‐15 assay have been previously described [14]. An internal study confirmed that the reference values provided by the manufacturer were valid for our study population.

### 2.4 MRI Acquisition

We prospectively studied with MR imaging 49 patients between May 2023 and May 2025. Images were acquired on a 1.5 Tesla Philips muscle magnetic image (MRI) system (Achieva, release 5.7.1; Philips Medical Systems, Eindhoven, The Netherlands).

A dedicated Muscle MRI of both the thighs and legs (separated scans) was performed in all patients. MRI was performed with a standard anterior 16 channel sense‐XL‐torso coil and the built‐in Q body coil.

The standardised MRI scanning protocol included conventional axial T1‐weighted sequence (TR = 423 ms, TE = 15 ms) and axial short tau inversion recovery (STIR) sequence (TR = 4102, TI = 150, effective TE = 30, TSE factor = 15), as well as coronal proton density with fat saturation (PDFS) sequence (TR = 2225, TE = 70, TSE factor = 18) of both the thighs and legs.

Axial 6‐echo 3D fast field echo (FFE) Dixon sequence (mDIXON‐QUANT) of the thighs was additionally acquired with the following parameters: repetition time/echo time fixed to the shortest, voxel size = 1.6 × 1.6 × 3 mm and field of view (FOV) 520 × 340 × 300 mm. To avoid T1 saturation, a low flip angle of only 5° was applied 0.4 image types were generated. The water, fat, fat fraction and T2* images.

### 2.5 MRI Analysis

#### 2.5.1 Semi‐Quantitative Assessment

Two experienced radiologists (ABM, VGM), blinded to the genetic diagnosis and clinical data, evaluated the images using the institutional Picture Archiving and Communication System (PACS) viewer.

Muscles from pelvis, thighs and lower legs were scored by consensus in axial T1‐sequences with the semiquantitative Mercuri visual scale (MVS) modified by Fisher: (0) normal appearance; (1) mild involvement, less than 30% of individual muscle volume; (2) moderate involvement, 30%–60% of individual muscle volumes; (3) severe involvement, > 60% of individual muscle; (4) end stage, all the muscle is severely affected, replaced by connective tissue and fat.

The muscles were scored on the right side, but any asymmetries were recorded. The following muscles were scored: *gluteus maximus*, *gluteus medius*, *gluteus minor*, *pectineus*, *tensor fascia latae*, *rectus femoris*, *vastus intermedius*, *vastus lateralis*, *vastus medialis*, *adductor magnus*, *sartorius*, *gracilis*, *biceps femoris long head*, *semitendinosus* and *semimembranosus*.

#### 2.5.2 Quantitative Assessment

Segmentation of the same muscles that were analysed for semi‐quantitative assessment was performed in all patients in proximal, middle and distal regions of the right thigh. The segmentation was performed in axial slices of the fat fraction maps derived from the mDIXON QUANT acquisition using vendor's application (Philips portal).

Specifically, manual regions of interest (ROIs) were drawn by consensus in three selected axial slices of the obtained fat fraction maps based on anatomical landmarks, to separately enclose the outer contour of the different muscles (FIG) by ABM and VGM: (1) proximal slice at the level of the supra‐acetabular region: assessment of *gluteus medius* and *gluteus minor*; (2) middle slice at the level of the lesser trochanter: assessment of *gluteus maximus*, *tensor fascia lata* and *pectineus*; (3) distal slice at the level of femoral mid‐diaphysis: assessment of *rectus femoris*, *vastus intermedius*, *vastus lateralis*, *vastus medialis*, *adductor magnus*, *sartorius*, *gracilis*, *biceps femoris long head*, *semitendinosus* and *semimembranosus*.

Although volumetric muscle segmentation would provide a more precise estimate of total muscle fat burden, this approach was not feasible due to the lack of dedicated automated or semi‐automated segmentation software compatible with the acquisition protocol. Manual volumetric segmentation was therefore considered impractical and potentially less reproducible in this cohort.

Muscle‐specific proton density fat fraction (PDFF) values were obtained from regions of interest (ROIs) in individual muscles. PDFF is defined as the ratio of the density of mobile protons from fat (triglycerides) to the total density of mobile protons from both fat and water, reflecting the fat content within the tissue. PDFF (hereafter referred to as FF) was selected as it represents a standardised, quantitative and highly reproducible MRI biomarker of muscle fat infiltration, largely independent of scanner settings and acquisition parameters [11, 12, 15, 16].

A total muscle FF score (FF sum) was calculated for each patient as the arithmetic sum of fat fraction values across all evaluated muscles, following the summative approach previously applied by Wang and colleagues in FSHD [17]. The FF sum was conceived as a non‐volume‐weighted composite index reflecting the overall burden of structural muscle involvement, rather than as a volumetric estimate of total muscle fat mass. The overall fat fraction for the entire cohort, considering all muscles in all patients, was also recorded. Mean FF values per muscle were calculated to explore patterns of muscle involvement and to compare across genotypes.

### 2.6 Statistical Analysis

Descriptive statistics were used to summarise demographic, clinical, laboratory, functional and imaging variables. Continuous variables were expressed as mean and standard deviation (SD) when normally distributed, or as median and interquartile range (IQR) otherwise. Categorical variables were summarised as counts and percentages.

Correlations between total lower limb muscle fat fraction (FF sum) and motor function measures, laboratory biomarkers and disease duration were assessed using Spearman's rank correlation coefficient. These analyses were pre‐specified, hypothesis‐driven correlations grounded on biological plausibility and prior evidence linking muscle fat infiltration with muscle weakness and functional impairment in other genetic muscle disorders. Because each correlation tested a distinct, biologically motivated a priori hypothesis rather than constituting a screening of unselected endpoints, no additional correction for multiple testing was applied to these correlation analyses. Correlation strength was interpreted according to conventional thresholds, as commonly applied in clinical imaging studies. Group comparisons across genotypes and phenotypes were performed using non‐parametric tests (Kruskal–Wallis test with post hoc pairwise comparisons when appropriate), with correction for multiple comparisons applied to the post hoc contrasts. Agreement between quantitative muscle fat fraction values and Mercuri visual scores was assessed using Spearman correlation coefficients and intraclass correlation coefficients (ICCs) based on a two‐way mixed‐effects model for absolute agreement.

Multiple correspondence analysis (MCA) was applied to categorical muscle MRI variables derived from Mercuri scores to explore patterns of muscle involvement, with genotype or phenotype included as supplementary variables for visualisation. MCA was used as an exploratory multivariate approach to visualise patterns of muscle involvement. No inferential conclusions were derived from MCA results. The consistency between quantitative MRI metrics, semi‐quantitative visual scores and independent clinical and functional outcome measures was considered an internal validation of the imaging biomarkers. Statistical significance was set at *p* < 0.05. All analyses were conducted using Stata Statistical Software for Windows, version 16 (StataCorp. 2019. Stata Statistical Software: Release 16. College Station, Texas: StataCorp LLC). Graphs were generated using GraphPad Prism, version 10.4.2.

## 3 Results

### 3.1 Cohort Characteristics

The study included 49 adult patients with mitochondrial myopathies, of whom 25 (51.0%) were female. The mean age at disease onset was 26.3 ± 15.1 years, and the mean age at the time of muscle MRI was 50.0 ± 12.3 years. The median disease duration at evaluation was 23 years (IQR 15–31). The demographic, genetic, clinical, laboratory and functional characteristics of the cohort are summarised in Table 1.

**TABLE 1:** Demographic, genetic, clinical, laboratory and functional characteristics of the study cohort.

ID	Age at MRI	Age at onset	Duration of the disease	Gen	Variant 1	Variant 2	Phenotype	CK	Creatinine	GDF15	MRC	6MWT	100 MRT	NSAA
1	40	18	22	*TK2*	c.604_606delAAG	c.604_606delAAG	Progressive myopathy	365	0.32	1922	25	595	41	31
2	47	31	16	*TK2*	c.604_606delAAG	c.604_606delAAG	Progressive myopathy	245	0.34	3189	NA	460.5	73	25
3	64	30	34	*TK2*	c.604_606delAAG	c.604_606delAAG	Progressive myopathy	195	0.37	3774	17	432	76	20
4	43	18	25	*TK2*	c.604_606delAAG	c.623A>G	Progressive myopathy	1587	0.33	3635	19.5	517	63	25
5	53	45	8	*TK2*	c.604_606delAAG	c.623A>G	PEO PLUS	402	0.54	1789	24	642.5	34	33
6	70	40	30	*TK2*	c.604_606delAAG	c.604_606delAAG	PEO PLUS	684	0.54	2192	20.5	NA	NA	10
7	32	16	16	*TK2*	c.323C>T	c.323C>T	Exercise intolerance	615	0.61	1718	NA	483.5	65	NA
8	50	40	10	*TK2*	c.604_606delAAG	c.604_606delAAG	Exercise intolerance	1612	0.5	1856	25	478	53	34
9	45	20	25	*POLG*	c.2864A>G		Progressive myopathy	240	0.75	4533	18	508	37	33
10	55	40	15	*POLG*	c.2864A>G		PEO PLUS	94	0.63	2079	NA	375	NA	28
11	62	34	28	*POLG*	c.2864A>G		PEO PLUS	157	0.43	5996	18	NA	NA	10
12	60	45	15	*POLG*	c.752C>T+c.1760C>T	c.2537C>T	PEO PLUS	132	0.61	4425	24	334	85	33
13	45	30	15	*POLG*	c.2573C>T		Exercise intolerance	355	0.95	973	25	400	47	34
14	57	35	22	*POLG*	c.2537C>T	c.3680_3684del	PEO PLUS	94	0.45	2312	17	259	100	15
15	56	55	1	*POLG*	c.2573T		HiperCKemia	510	0.78	1880	NA	400	48	NA
16	59	35	24	*POLG*	c.1760C>T	c.752C>T	Other	246	0.65	5322	25.5	NA	NA	16
17	68	59	9	*TWNK*	c.1361T>G		PEO PLUS	68	1.32	2612	24	371	91	34
18	61	5	56	*TWNK*	c.1084G>C		PEO Pure	77	0.69	1326	25	353	93	34
19	32	17	15	*TWNK*	c.1121G>A		PEO Pure	394	0.86	2351	24	460	28	34
20	78	63	15	*TWNK*	c.1070G>C		PEO Pure	139	0.81	1529	25	499	62	34
21	42	16	26	mtDNA	Single deletion		PEO Pure	121	0.44	8776	17	NA	NA	NA
22	65	5	60	mtDNA	Single deletion		PEO PLUS	81	0.69	2584	16.5	NA	NA	NA
23	53	16	37	mtDNA	Single deletion		PEO PLUS	273	0.44	6473	20	367.5	68	26
24	53	15	38	mtDNA	Single deletion		PEO PLUS	314	0.47	3722	22	420	58	32
25	45	5	40	mtDNA	Single deletion		PEO PLUS	NA	NA	6172	17	202	130	19
26	49	15	34	mtDNA	Single deletion		PEO PLUS	137	0.57	1789	14.5	259	150	24
27	48	25	23	mtDNA	Single deletion		PEO Pure	100	0.79	2228	25	500	47	34
28	28	20	8	mtDNA	Single deletion		PEO Pure	977	0.65	1769	25	500	42	34
29	42	20	22	mtDNA	Single deletion		PEO Pure	1879	0.65	2238	25	600	31	34
30	41	17	24	mtDNA	Single deletion		PEO Pure	276	0.9	3233	25	692	24	34
31	36	13	23	mtDNA	Single deletion		PEO Pure	281	0.57	2166	25	NA	NA	34
32	34	25	9	mtDNA	Single deletion		PEO Pure	137	0.84	2745	25	549	36	34
33	64	30	34	mtDNA	Single deletion		PEO Pure	69	0.69	3129	25	515.5	46	34
34	53	16	37	mtDNA	Single deletion		PEO Pure	NA	NA	NA	24	675	35	34
35	62	40	22	mtDNA	Single deletion		PEO Pure	279	0.67	3302	25	518	45	31
36	59	32	27	mtDNA	Single deletion		PEO Pure	431	11	3299	25	411	53	32
37	39	12	27	mtDNA	Single deletion		PEO Pure	73	0.68	3952	25	599	29	34
38	49	16	33	mtDNA	Single deletion		PEO Pure	139	1.2	3141	25	545	25	34
39	28	15	13	mtDNA	Single deletion		PEO Pure	179	0.82	3262	25	350	61	31
40	41	16	25	mtDNA	m.3243A>G, MTTL1		Progressive myopathy	164	0.61	2870	21.5	547	49	NA
41	60	50	10	mtDNA	m.8344A>G, MTTK		Progressive myopathy	240	0.49	7571	24.5	412.5	80	29
42	65	50	15	mtDNA	m.3243A>G, MTTL1		Progressive myopathy	304	0.44	8228	23	437	74	29
43	25	5	20	mtDNA	m.3243A>G, MTTL1		PEO Plus	127	0.85	2427	24.5	432	37	NA
44	32	8	24	mtDNA	m.3243A>G, MTTL1		PEO Plus	505	0.75	3274	25	585	28	33
45	51	30	21	mtDNA	m.4270T>C, MT‐TI		PEO Plus	196	1.12	2763	25	NA	NA	34
46	39	20	19	mtDNA	m.5992G>A		Exercise intolerance	141	0.73	1577	NA	435	36	34
47	50	18	32	mtDNA	m.5692T>C, MTTN		Exercise intolerance	251	0.71	2710	22.5	186	187	20
48	58	49	9	mtDNA	m.8344A>G, MTTK		Exercise intolerance	NA	NA	NA	NA	NA	NA	NA
49	60	15	45	mtDNA	m.3243A>G, MTTL1		Other	124	16	2194	25	445	50	34

*Note:* The table summarises individual patient data including age at MRI, age at disease onset, disease duration, genetic diagnosis and variants, clinical phenotype, laboratory biomarkers (creatine kinase [CK], serum creatinine and growth differentiation factor 15 [GDF15]), global muscle strength assessed by the Medical Research Council (MRC) score and functional outcomes (6‐min walk test [6MWT], 100‐m run test [100MRT] and North Star Ambulatory Assessment [NSAA]). Missing data are indicated as NA.

### 3.2 Genetic and Clinical Features

Pathogenic variants in mtDNA were identified in 29 patients (59.1%), including 10 patients (20.4%) with point mutations and 19 patients (38.8%) with single large‐scale mtDNA deletions. Nuclear gene variants were present in 20 patients, including *POLG* (*n* = 8), *TK2* (*n* = 8) and *TWNK* (*n* = 4).

Clinically, seven patients (14.3%) presented with exercise intolerance without overt muscle weakness. Progressive external ophthalmoplegia (PEO) was observed in 32 patients (65.3%), half of whom had isolated PEO and half had PEO associated with extraocular muscle weakness (PEO‐plus). Eight patients (16.3%) presented with a phenotype dominated by progressive myopathy.

Distinct genotype–phenotype associations were observed. Single large‐scale mtDNA deletions were most frequently associated with pure PEO, whereas progressive myopathy was most commonly observed in patients with *TK2* variants. *POLG* variants were predominantly associated with PEO‐plus phenotypes, while *TWNK* variants were mainly associated with pure PEO.

### 3.3 Laboratory Biomarkers and Motor Function

Median serum creatine kinase (CK) levels were 240 IU/L (IQR 132–365). Mean serum creatinine was 0.68 ± 0.23 mg/dL, and median GDF15 concentration was 2745 pg./mL (IQR 2079–3722).

Global muscle strength assessed by the total Medical Research Council (MRC) score showed a median value of 24.5 (IQR 20.5–25). Functional assessments revealed a median 6‐min walk distance of 460 m (IQR 400–518), a median 100‐m run test completion time of 49.5 s (IQR 36.5–73.5) and a median North Star Ambulatory Assessment (NSAA) score of 33 (IQR 26–34).

### 3.4 Muscle MRI Fat Fraction and Visual Grading

**FIGURE 1:**
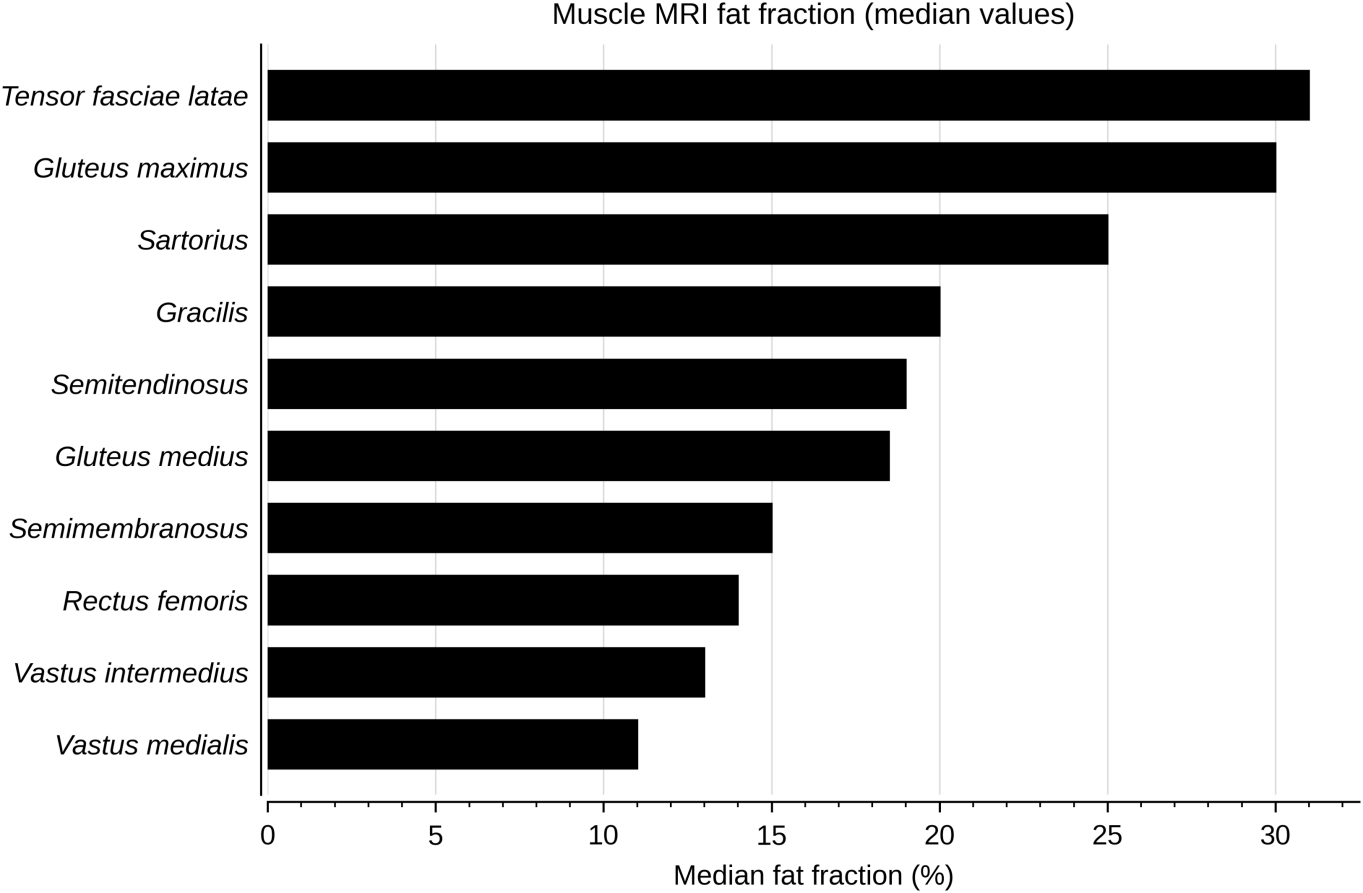
Distribution of muscle fat fraction across lower limb muscles. Bar plot showing median proton density fat fraction (PDFF) values for individual lower limb muscles assessed by quantitative muscle MRI. Muscles are ordered from highest to lowest median fat fraction, highlighting preferential patterns of muscle involvement. The distribution illustrates greater fatty replacement in proximal and selective thigh muscles, with relative sparing of quadriceps muscles.

Quantitative muscle MRI of the lower limbs revealed heterogeneous patterns of fatty replacement across individual muscles. The highest median fat fraction (FF) values were observed in *tensor fasciae latae* (31% [IQR 18–41]), *gluteus maximus* (30% [IQR 20–49]), *sartorius* (25% [IQR 18–40]) and *gracilis* (20% [IQR 13–28]). Intermediate involvement was observed in *semitendinosus* (19% [IQR 12–27]), *gluteus medius* (18.5% [IQR 16–22]), *semimembranosus* (15% [IQR 12–22]) and *rectus femoris* (14% [IQR 12–19]). Within the *quadriceps*, lower fat fraction values were observed in *vastus intermedius* (13% [IQR 11–16]) and *vastus medialis* (11% [IQR 9–14]), Figure 1.

**FIGURE 2:**
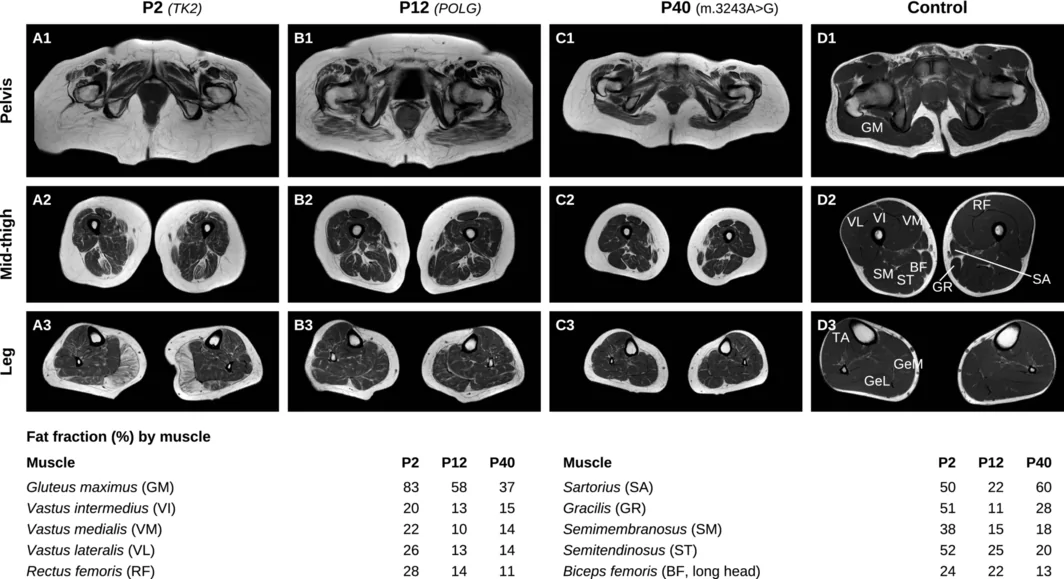
Axial muscle MRI at three anatomical levels in three patients and an unaffected control. Columns correspond to individual participants: (A) P2, *TK2*; (B) P12, *POLG*; (C) P40, m.3243A > G; (D) unaffected control. Rows correspond to the three standardised levels: (1) pelvis, (2) mid‐thigh, (3) leg. Images are axial T1‐weighted. Muscles are labelled on the control panels and abbreviated as follows: GM, *gluteus maximus*; VI, *vastus intermedius*; VM, *vastus medialis*; VL, *vastus lateralis*; RF, *rectus femoris*; SA, *sartorius*; GR, *gracilis*; SM, *semimembranosus*; ST, *semitendinosus*; BF, *biceps femoris* (long head); TA, *tibialis anterior*; GeM, *gastrocnemius medialis*; GeL, *gastrocnemius lateralis*. The leg muscles labelled in D3 are shown for anatomical orientation only and were not quantified. The table gives the fat fraction (%) of the 10 pelvic‐girdle and thigh muscles quantified in each patient.

Representative axial images at the three standardised levels in three patients with different genotypes and in an unaffected control, together with the fat fraction of the quantified muscles, are shown in Figure 2.

A strong and statistically significant correlation was observed between quantitative fat fraction values and Mercuri visual scores across analysed muscles (*r* > 0.7, *p* < 0.00001). In genotypic subgroups with smaller sample sizes (*POLG*, *TK2*, *TWNK*), intraclass correlation coefficients (ICCs) reached 1.00, reflecting excellent agreement between both evaluation methods. These values should be interpreted with caution, as they are influenced by the small sample sizes and the limited variability within these subgroups.

### 3.5 Correlations Between Muscle Fat Fraction, Motor Function and Circulating Biomarkers

**FIGURE 3:**
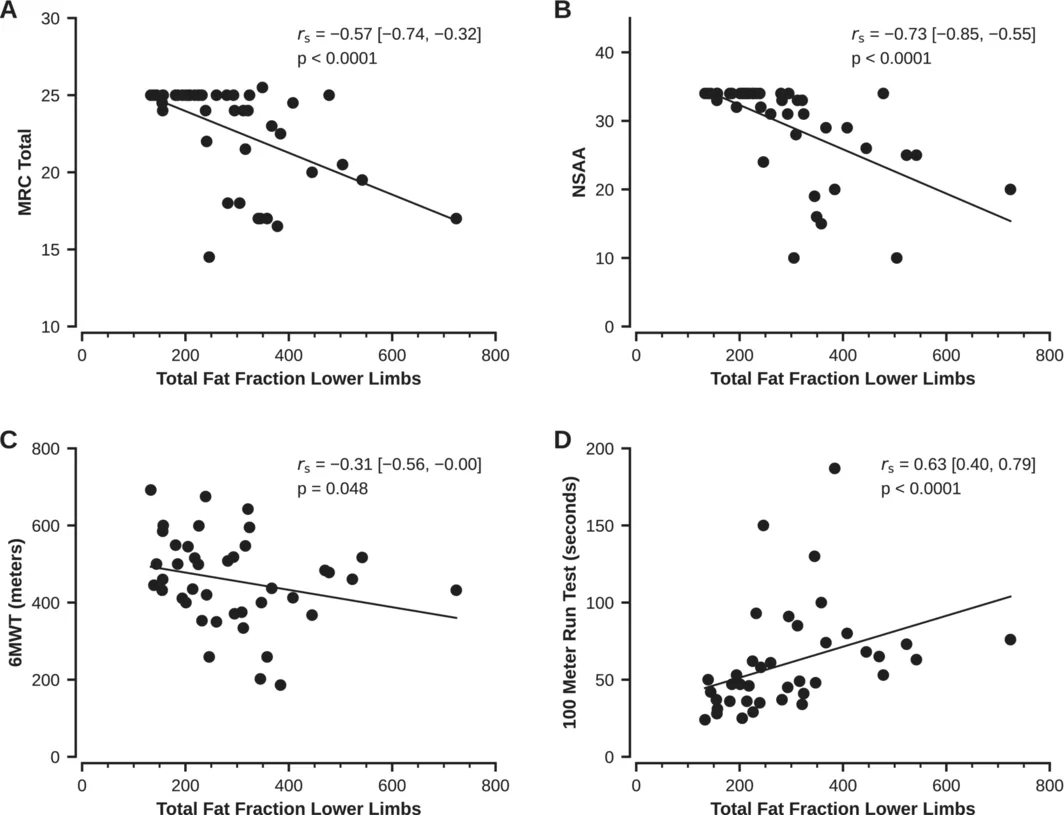
Association between lower limb muscle fat fraction and motor function outcomes. Scatter plots showing the relationship between total lower limb muscle fat fraction and (A) global muscle strength assessed by the total Medical Research Council (MRC) score, (B) functional performance assessed by the North Star Ambulatory Assessment (NSAA), (C) walking distance in the 6‐min walk test (6MWT) and (D) performance time in the 100‐m run test (100MRT). Spearman's rank correlation coefficients (*r*), 95% confidence intervals and *p* values are indicated for each analysis. Higher muscle fat fraction was associated with reduced muscle strength and functional performance, and with longer completion times in timed motor tests. Solid lines represent linear regression fits for visualisation purposes.

FF sum showed a strong inverse correlation with global muscle strength assessed by the total MRC score (*r* = −0.567, 95% CI −0.745 to −0.314, *p* < 0.0001), indicating greater muscle weakness in patients with higher fat infiltration (Figure 3A).

Similarly, FF sum correlated strongly and inversely with functional performance measured by the NSAA (*r* = −0.731, 95% CI −0.849 to −0.542, *p* < 0.0001) (Figure 3B). A positive correlation was observed between FF sum and 100MRT completion time (*r* = 0.629, 95% CI 0.387 to 0.790, *p* < 0.0001) (Figure 3C), consistent with worse motor performance in patients with higher fat fraction. A weak inverse correlation was observed between FF sum and 6MWT distance (*r* = −0.311, 95% CI −0.571 to 0.005, *p* = 0.0476) (Figure 3D).

**FIGURE 4:**
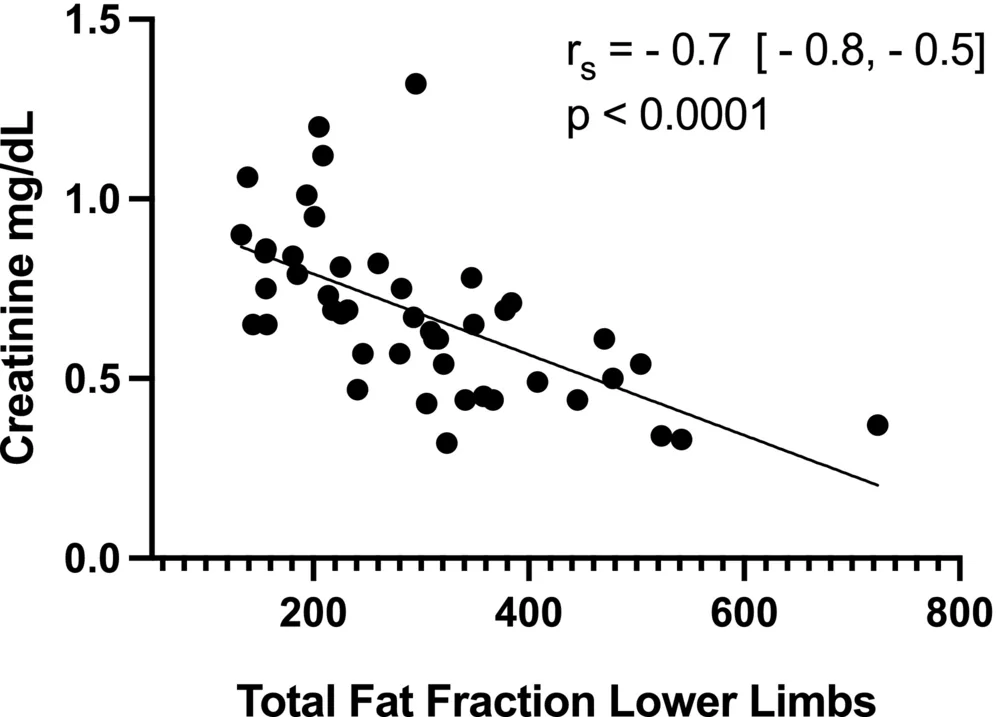
Association between total muscle fat fraction and serum creatinine levels. Scatter plot showing the relationship between total lower limb muscle fat fraction (FF sum) and serum creatinine concentration. A strong inverse association was observed, indicating lower creatinine levels in patients with higher muscle fat infiltration. Spearman's rank correlation coefficient (*r*), 95% confidence interval and *p* value are shown. The solid line represents the linear regression fit for visualisation purposes.

Regarding circulating biomarkers, FF sum showed a strong inverse correlation with serum creatinine levels (*r* = −0.715, 95% CI −0.835 to −0.529, *p* < 0.0001) (Figure 4). No significant correlations were observed between FF sum and CK levels (*r* = 0.142, *p* = 0.347) or GDF15 concentrations (*r* = 0.244, *p* = 0.098).

Overall, these results indicate that quantitative muscle MRI‐derived fat fraction is closely associated with both strength and functional impairment, as well as with biomarkers reflecting muscle mass, supporting its value as an imaging biomarker of disease severity in this cohort.

Disease duration was not significantly associated with global muscle strength (MRC score), functional performance measures (NSAA, 100MRT, or 6MWT), or total lower limb fat fraction.

### 3.6 Genotype‐Specific Differences

No significant differences were observed among genotypes with respect to age at MRI or disease duration. Patients with single large‐scale mtDNA deletions (*n* = 19) had a significantly younger age at disease onset compared with patients with nuclear gene variants, including *POLG* (*n* = 8; *p* < 0.001), *TK2* (*n* = 8; *p* = 0.010) and *TWNK* (*n* = 4; *p* = 0.031). No significant differences in age at onset were observed between patients with mtDNA deletions and those with mtDNA point mutations.

Serum creatinine levels were significantly lower in patients with TK2 deficiency compared with other genotypes (*p* < 0.003). Motor function measures did not differ significantly across genotypes, including MRC score, NSAA, 6MWT and 100MRT. A trend toward poorer motor performance and higher CK levels was observed in TK2‐related disease, although these differences did not remain significant after correction for multiple comparisons.

**FIGURE 5:**
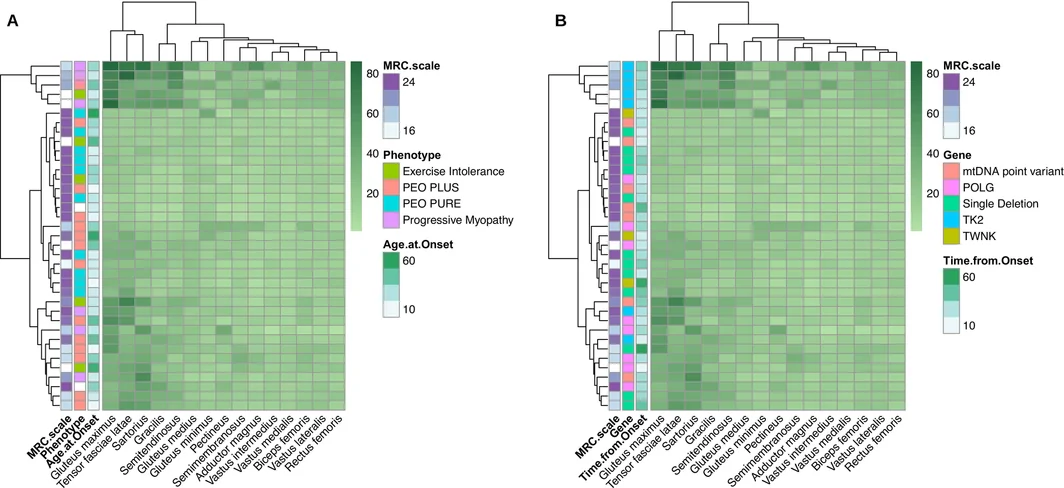
Heatmap representation of muscle fat fraction by phenotype and genotype. (A) Heatmap showing the distribution of muscle fat fraction across lower limb muscles according to clinical phenotype, including exercise intolerance, pure progressive external ophthalmoplegia (PEO), PEO‐plus and progressive myopathy. (B) Heatmap showing the distribution of muscle fat fraction according to genotype, including mtDNA point variants, single large‐scale mtDNA deletions and pathogenic variants in *POLG*, *TK2* and *TWNK*. In both panels, rows represent individual patients and columns correspond to specific muscle groups. Hierarchical clustering was performed based on muscle fat fraction values. Annotation bars display clinical variables including phenotype or genotype, age at disease onset, time from disease onset and total Medical Research Council (MRC) score.

Quantitative MRI demonstrated significantly higher thigh muscle fat fraction in patients with *TK2* variants, with greater involvement of the gluteus maximus, gracilis and semitendinosus muscles (*p* < 0.001). These genotype‐specific patterns of muscle involvement are further illustrated by heatmap‐based visualisation of muscle fat fraction across individual muscles and patients (Figure 5B).

Table 2 summarises the genotype‐based comparison of demographic, clinical, functional, laboratory and muscle MRI characteristics.

**TABLE 2:** Genotype‐based comparison of demographic, clinical, functional, laboratory and muscle MRI characteristics.

	mtDNA variant	Large‐scale single deletion	*POLG*	*TK2*	*TWNK*	*p*
	*n* = 10	*n* = 19	*n* = 8	*n* = 8	*n* = 4	
**Age at MRI**	48.10 (13.35)	46.84 (11.22)	54.88 (6.49)	49.88 (12.46)	59.75 (19.77)	0.26
**Age at disease onset**	26.10 (17.58)	**18.58 (8.71)**	36.75 (10.36)	29.75 (11.40)	36 (29.33)	0.020
**Disease duration**	20.50 (15–25)	27 (22–37)	18.50 (15–24.50)	19 (13–27.50)	15 (12–35.50)	0.21
**Phenotype**						**<0.001**
**Exercise Intolerance**	3 (30%)	0 (0%)	2 (25%)	2 (25%)	0 (0%)	
**PEO PLUS**	3 (30%)	6 (31.58%)	**4 (50%)**	2 (25%)	1 (25%)	
**PEO PURE**	0 (0%)	**13 (68.42%)**	0 (0%)	0 (0%)	**3 (75%)**	
**Progressive myopathy**	3 (30%)	0 (0%)	1 (12.50%)	**4 (50%)**	0 (0%)	
**CK activity**	196 (141–251)	179 (121–281)	198.50 (113–300.50)	508.50 (305–1135.50)	108 (72.50–266.50)	0.042
**Creatinine levels**	0.75 (0.23)	0.71 (0.20)	0.66 (0.17)	**0.44 (0.12)**	0.92 (0.28)	**0.003**
**GDF15**	2763 (2427–3274)	3187 (2238–3722)	3368.50 (1979–4927)	2057 (1822–3412)	1940 (1427–2481)	0.33
**MRC total**	24.50 (22.75–25)	25 (20–25)	21 (18–25)	22.25 (19.50–25)	24.50 (24–25)	0.79
**6MWT**	436 (422.25–496)	507.75 (389.25–574)	387.50 (334–400)	483.50 (460.50–595)	415.50 (362–479.50)	0.11
**100 MRT**	49.50 (36.50–77)	45.50 (33–59.50)	48 (47–85)	63 (41–73)	76.50 (45–92)	0.60
**NSAA**	33 (29–34)	34 (31–34)	28 (15–33)	25 (20–33)	34 (34–34)	0.023
***Gluteus maximus* FF (%)**	21 (18–39)	24 (19–36)	33 (27–39)	**65 (49.50–75.50)**	22.50 (18–29)	**<0.001**
***Gluteus medius* FF (%)**	16.50 (14.50–18.50)	18.50 (16–21.50)	20 (17–22)	20 (17–36)	17 (15–20)	0.14
***Tensor fasciae latae* FF (%)**	19 (16–35)	27 (16–41)	34 (21–38.50)	43 (40–60)	24.50 (10–34.50)	0.027
***Vastus intermedius* FF (%)**	11.50 (10–15)	13 (11–15)	14 (13–16)	15.50 (11–28)	10.50 (10–14)	0.45
***Vastus medialis* FF (%)**	12 (9–14)	10 (9–12)	11.50 (10–14.50)	17 (14.50–23.50)	9.50 (8–10.50)	0.038
***Vastus lateralis* FF (%)**	12.50 (10–15)	13 (11–18)	15.50 (13–18)	25 (17.50–27.50)	16 (13–16)	0.093
***Rectus femoris* FF (%)**	14 (11–18)	12 (11–15)	13.50 (11.50–15)	27 (19–31)	19.50 (15–21)	0.004
***Sartorius* FF (%)**	19.50 (10–33)	24 (18–30)	37.50 (19.50–46.50)	49.50 (31.50–58.50)	17.50 (15–20.50)	0.010
***Gracilis* FF (%)**	14 (8–23)	18 (12–24)	20 (12.50–28.50)	**44.50 (35.50–51)**	15.50 (11–18)	**<0.001**
***Semimembranosus* FF (%)**	11.50 (6–19)	14 (11–15)	19.50 (14–28)	23.50 (18.50–34)	16 (13–20)	0.022
***Semitendinosus* FF (%)**	16.50 (7–22)	17 (11–25)	21 (13.50–26)	**54 (34.50–61)**	11.50 (11–15.50)	**<0.001**
**Total FF (%)**	217.00 (156.00–367.00)	239.00 (185.00–293.00)	310.50 (293.50–348.00)	**491.00 (397.00–532.50)**	228.50 (190.50–263.50)	**0.001**

*Note:* Demographic variables, clinical phenotypes, laboratory biomarkers, motor function measures and quantitative muscle MRI fat fraction (FF) values are shown according to genetic diagnosis, including mtDNA point mutations, single large‐scale mtDNA deletions and pathogenic variants in *POLG*, *TK2* and *TWNK*. Continuous variables are expressed as mean (standard deviation) or median (interquartile range), as appropriate and categorical variables as number (percentage). *p* Values correspond to between‐group comparisons across genotypes. Bold values indicate groups showing differences in post hoc pairwise comparisons. Overall group comparisons were performed using Kruskal–Wallis tests. Pairwise post hoc comparisons were performed using Dunn's test with Bonferroni correction.

Abbreviations: PEO, progressive external ophthalmoplegia; CK, creatine kinase; GDF15, growth differentiation factor 15; MRC, Medical Research Council; 6MWT, 6‐min walk test; 100MRT, 100‐m run test; NSAA, North Star Ambulatory Assessment; FF, fat fraction.

### 3.7 Phenotype‐Specific Muscle Involvement

**FIGURE 6:**
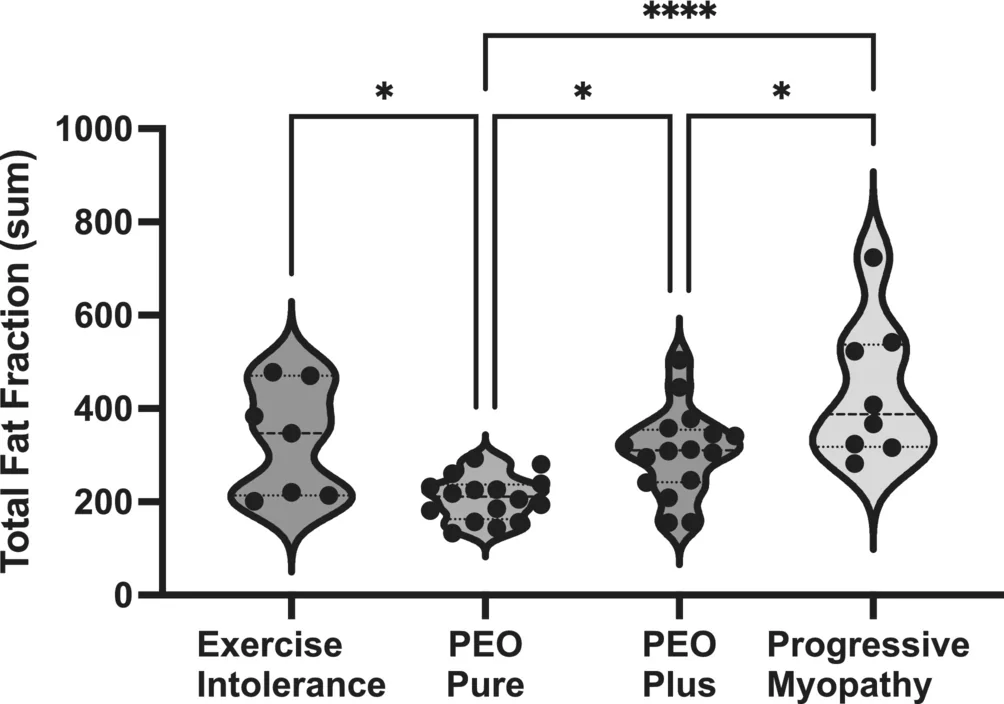
Differences in total muscle fat fraction across clinical phenotypes. Violin plots showing the distribution of total lower limb muscle fat fraction (FF sum) across clinical phenotypes, including exercise intolerance, pure progressive external ophthalmoplegia (PEO), PEO‐plus and progressive myopathy. Individual data points are shown. Significant differences between groups are indicated (*p < 0.05; ****p < 0.0001).

Patients presenting with a progressive myopathy phenotype (*n* = 8) showed significantly greater fatty replacement compared with other phenotypes (*p* < 0.001), including PEO‐plus (*n* = 16). A significant difference was also observed between patients with isolated PEO (*n* = 16) and those with PEO associated with extraocular muscle weakness (*n* = 16; *p* = 0.001). Patients classified as having exercise intolerance (*n* = 7) exhibited significantly greater fatty replacement compared with those with pure PEO (*n* = 16; *p* = 0.033), indicating subclinical muscle involvement despite preserved strength (Figure 6).

Heatmap representations based on quantitative muscle fat fraction further highlighted phenotype‐specific patterns of muscle involvement, revealing a gradient of disease severity and selective muscle involvement across phenotypic groups (Figure 5A).

### 3.8 Multivariate Analysis of Muscle MRI Patterns

MCA of Mercuri‐based categorical muscle MRI variables revealed consistent patterns of muscle involvement. In both phenotype‐ and genotype‐based analyses, Dimension 1 (Dim‐1) explained approximately 18%–19% of total inertia and represented a gradient of global disease severity, driven by increasing contributions from higher Mercuri grades across multiple muscles, particularly *quadriceps*, *tensor fasciae latae*, *sartorius*, *gracilis* and *gluteus maximus*. Dimension 2 (Dim‐2), explaining approximately 9%–10% of inertia, captured a more selective pattern involving *rectus femoris*, *gracilis* and *gluteus maximus*.

In phenotype‐based analyses, patients with progressive myopathy clustered toward positive values of Dim‐1, while patients with pure PEO clustered toward negative values, with PEO‐plus phenotypes occupying intermediate positions. In genotype‐based analyses, patients with *TK2* variants clustered toward higher values of both dimensions, whereas patients with single large‐scale mtDNA deletions clustered toward lower severity regions. *POLG* and *TWNK* cases showed intermediate and overlapping distributions, reflecting substantial intragroup heterogeneity.

## 4 Discussion

In this study, we demonstrate that quantitative muscle MRI provides clinically meaningful information in adults with primary mitochondrial myopathies, capturing disease severity across a heterogeneous spectrum of genotypes and phenotypes. Using FF as an objective and continuous measure of muscle degeneration [11], we show that structural muscle involvement correlates closely with muscle strength, functional performance and muscle mass, supporting its role as a robust imaging biomarker. These findings extend the utility of muscle MRI to mitochondrial myopathies.

Muscle MRI is well established as a key diagnostic and prognostic tool in a wide range of genetically determined muscle diseases. In dystrophinopathy [7, 18], limb‐girdle muscular dystrophies [6], congenital myopathies [19] and other inherited disorders [20, 21], characteristic patterns of muscle involvement identified by MRI have proven valuable for differential diagnosis, guiding genetic testing and monitoring disease progression. In these conditions, the extent and distribution of fatty replacement correlate closely with muscle strength, functional performance and clinical staging, and quantitative MRI techniques such as Dixon‐based fat fraction measurements have emerged as sensitive and reproducible outcome measures [18, 22, 23].

In contrast, the role of muscle MRI in mitochondrial myopathies has historically been less clearly defined, with only single cases or small series having been published to date [24, 25]. Previous studies have emphasised that patients with primary mtDNA mutations, particularly those presenting with chronic PEO, may show normal or near‐normal MRI findings in limb muscles, limiting the diagnostic utility of imaging in some clinical contexts [26]. As a result, muscle MRI has traditionally been considered less informative in mitochondrial disease than in other inherited myopathies, except in selected nuclear‐encoded disorders [10].

The present study adds to the growing body of evidence supporting a more prominent role for muscle MRI in mitochondrial myopathies [4], particularly when quantitative approaches are applied. By using MRI‐derived fat fraction (PDFF) as a continuous measure of muscle degeneration, this work extends earlier semi‐quantitative observations [10] and demonstrates that structural muscle involvement can be robustly quantified and meaningfully related to clinical status. The strong associations observed between total lower limb fat fraction and muscle strength (assessed with the MRC score), functional performance (NSAA and 100MRT) and serum creatinine levels (together with a weaker association with the 6MWT) parallel findings reported in other genetic myopathies and support the validity of FF as an imaging biomarker in this setting.

Although a prospectively recruited age‐matched healthy control cohort was not available in this study, the fat fraction values observed in our patients clearly exceed those reported for healthy adults in the published literature. In healthy adults of comparable age, quadriceps PDFF values typically range from 2% to 4% and hamstring values from 3% to 5%, with only modest age‐related increases [27, 28]. By contrast, even the least affected muscles in our cohort (vastus medialis, vastus intermedius) showed median fat fractions of 11% and 13%, with markedly higher values in tensor fasciae latae (31%), gluteus maximus (30%), sartorius (25%) and gracilis (20%). Although direct comparison with published reference values is limited by differences in scanner platform, sequence implementation and segmentation strategy across studies [29, 30, 31], the magnitude of this difference supports that the fatty replacement observed in our cohort reflects disease‐related structural changes rather than physiological aging.

Importantly, our results help refine the understanding of genotype‐specific imaging patterns in mitochondrial disease. Consistent with previous reports [10, 32], patients with *TK2* deficiency exhibited the most severe and selective pattern of muscle involvement, with prominent fatty replacement affecting the pelvic girdle and antero‐medial thigh muscles. The concordance between qualitative pattern recognition described in earlier studies and the quantitative fat fraction data presented here reinforces the concept of a characteristic radiological signature for late‐onset *TK2* deficiency and demonstrates that this signature can be objectively quantified. In contrast, patients with single large‐scale mtDNA deletions tended to show milder limb muscle involvement, aligning with prior observations that limb MRI may appear relatively preserved in phenotypes dominated by PEO [26].

Beyond established phenotypes, our findings highlight the ability of quantitative muscle MRI to detect subclinical muscle involvement. Patients classified as having exercise intolerance, despite the absence of overt muscle weakness, showed higher levels of muscle fat infiltration than those with pure PEO. This extends previous observations by demonstrating that quantitative MRI may detect structural muscle abnormalities in patients without overt muscle weakness on routine clinical examination [23], supporting its potential value for detecting clinically subtle muscle involvement, as well as for patient stratification and longitudinal monitoring.

Notably, disease duration did not show significant associations with motor function, global muscle strength or total lower limb fat fraction. This suggests that the extent of muscle fat infiltration may be more closely related to the underlying genetic defect and clinical phenotype than to disease duration alone, underscoring the relevance of imaging‐based biomarkers over time‐based metrics in this heterogeneous group of disorders.

Previous MRI studies of extraocular muscles have shown that increased T1‐weighted signal intensity and T2 prolongation correlate with the severity of ophthalmoparesis, supporting the use of MRI‐based biomarkers to assess disease progression in PEO and to aid in the differential diagnosis between hereditary and acquired conditions [26, 33]. Although our study focuses on quantitative MRI of lower limb muscles, these observations are conceptually relevant, as they reinforce the ability of muscle MRI to capture disease‐specific patterns of involvement across different muscle groups. Taken together with our findings, this supports the broader value of muscle MRI for disease characterisation and severity assessment in mitochondrial myopathies.

In addition, muscle fat fraction showed a strong inverse association with serum creatinine, whereas no significant correlation was observed with creatine kinase or GDF15. This supports muscle fat fraction as a marker of structural muscle loss that overlaps with creatinine as a surrogate of muscle mass [34], while providing complementary information to circulating biomarkers [35]. In contrast, creatine kinase activity is more closely related to ongoing muscle damage, and increased GDF15 levels likely reflect a more nonspecific systemic response to mitochondrial dysfunction. Quantitative muscle MRI therefore captures the downstream consequence of muscle fibre loss and fatty replacement, providing a direct measure of chronic structural involvement.

Serum creatinine is also influenced by age, sex and renal function: Age‐related sarcopenia and sex‐related differences in muscle mass introduce physiological variation in creatinine values independent of disease state, while reduced glomerular filtration rate elevates serum creatinine for any given level of muscle mass. None of the patients in our cohort had impaired renal function, and the low mean serum creatinine values observed (0.68 ± 0.23 mg/dL) are consistent with reduced muscle mass as the primary biological driver of the observed inverse correlation. Larger studies with explicit adjustment for renal function and demographic covariates will nonetheless be needed to fully disentangle their relative contributions.

Taken together, these cross‐sectional findings position quantitative muscle MRI as a valuable complementary tool for the evaluation of mitochondrial myopathies. While MRI findings remain heterogeneous across genotypes and phenotypes, the use of fat fraction‐based metrics enables objective assessment of disease severity, facilitates genotype–phenotype correlations and enhances the detection of clinically silent muscle involvement. As disease‐modifying therapies continue to emerge—particularly for nuclear‐encoded mitochondrial disorders—quantitative muscle MRI may serve as a candidate biomarker for patient stratification and trial readiness. However, the cross‐sectional design of the present study does not allow assessment of longitudinal sensitivity to change or predictive validity, and prospective longitudinal studies will be required before PDFF can be adopted as a validated outcome measure in therapeutic trials [7].

Several limitations of the present study should be acknowledged. The relatively small cohort size, the heterogeneity of the genotype and phenotype subgroups, the cross‐sectional design and the focus on proximal lower limb muscles may limit the generalisability of our findings. Although individual muscle segmentation was feasible, volumetric analysis could not be performed due to the lack of automated or semi‐automated software compatible with our acquisition protocol, which restricted muscle‐specific correlations between fat fraction and clinical strength; the multiple correspondence analysis was also exploratory, with moderate inertia explained. The reported associations between fat fraction and clinical or laboratory variables rely on unadjusted bivariate analyses, pre‐specified on the basis of biological plausibility, as the sample size constraints noted above precluded an adequately powered multivariable adjustment for potential confounders such as age, sex, disease duration and genotype; disease duration itself did not show significant associations with fat fraction or functional measures, suggesting a modest contribution as a confounder, but the potential effects of age, sex and genotype cannot be formally excluded, and larger multicentre studies will be required to support multivariable modelling of these relationships.

A further limitation is the absence of a prospectively imaged, age‐matched healthy control group acquired with the same scanner platform and acquisition protocol. Although the magnitude of fat fraction values observed in our cohort substantially exceeds published reference values for healthy adults, the dependence of normal PDFF values on acquisition parameters, sequence implementation and segmentation strategy precludes the use of literature‐derived values as formal thresholds, and the establishment of locally acquired or harmonised multi‐centre normative reference values for quantitative muscle MRI remains a priority for future work in mitochondrial myopathies [36, 37]. Finally, longitudinal studies integrating quantitative MRI with functional outcomes will be needed to define the prognostic role of muscle fat fraction in mitochondrial myopathies and, in particular, to determine its sensitivity to change and its predictive validity as a candidate outcome biomarker in therapeutic trials.

## 5 Conclusions

Quantitative muscle MRI provides objective, clinically meaningful measures of muscle involvement in adults with primary mitochondrial myopathies. MRI‐derived fat fraction reflects disease severity across genotypes and phenotypes, correlates with strength, functional performance and muscle mass, and identifies clinically subtle muscle involvement. These cross‐sectional findings support PDFF as a candidate imaging biomarker for disease characterisation and patient stratification; longitudinal validation will be required to confirm its utility as an outcome biomarker in therapeutic trials.

## Funding

This work was supported by Instituto de Salud Carlos III PI22/01587 and Pretzel.

## Ethics Statement

This study has been approved by the appropriate institutional review board prior to initiation (N° CEIm: 22/493) and has been performed in accordance with the ethical standards laid down in the 1964 Declaration of Helsinki and its later amendments. All participants provided their written informed consent prior to their inclusion in the study.

## Conflicts of Interest

The authors declare no conflicts of interest.

## Data Availability

The data that support the findings of this study are available on request from the corresponding author. The data are not publicly available due to privacy or ethical restrictions.
